# Specific and Global RNA Regulators in *Pseudomonas aeruginosa*

**DOI:** 10.3390/ijms22168632

**Published:** 2021-08-11

**Authors:** Petra Pusic, Elisabeth Sonnleitner, Udo Bläsi

**Affiliations:** Max Perutz Labs, Department of Microbiology, Immunobiology and Genetics, Centre of Molecular Biology, Vienna Biocenter (VBC), University of Vienna, Dr. Bohrgasse 9/4, 1030 Vienna, Austria; petra.pusic@univie.ac.at (P.P.); elisabeth.sonnleitner@univie.ac.at (E.S.)

**Keywords:** *Pseudomonas aeruginosa*, base-pairing sRNAs, protein-binding RNAs, Hfq, Crc, RsmA, RsmN/F, post-transcriptional regulation

## Abstract

*Pseudomonas aeruginosa* (*Pae*) is an opportunistic pathogen showing a high intrinsic resistance to a wide variety of antibiotics. It causes nosocomial infections that are particularly detrimental to immunocompromised individuals and to patients suffering from cystic fibrosis. We provide a snapshot on regulatory RNAs of *Pae* that impact on metabolism, pathogenicity and antibiotic susceptibility. Different experimental approaches such as in silico predictions, co-purification with the RNA chaperone Hfq as well as high-throughput RNA sequencing identified several hundreds of regulatory RNA candidates in *Pae*. Notwithstanding, using in vitro and in vivo assays, the function of only a few has been revealed. Here, we focus on well-characterized small base-pairing RNAs, regulating specific target genes as well as on larger protein-binding RNAs that sequester and thereby modulate the activity of translational repressors. As the latter impact large gene networks governing metabolism, acute or chronic infections, these protein-binding RNAs in conjunction with their cognate proteins are regarded as global post-transcriptional regulators.

## 1. Introduction

High-throughput *RNA seq*uencing (RNAseq) has revolutionized the discovery of regulatory RNAs in bacteria [1,2,3,4,5,6,7,8,9,10,11,12,13]. These RNA regulators are mostly non-coding and usually synthesized under certain physiological conditions [3]. Once the trigger is lost, the regulatory RNA is often co-degraded with the target mRNA [14]. Regulatory RNAs are diverse and can act at all levels of gene expression, by (i) controlling DNA maintenance and silencing [15,16,17], (ii) activating or repressing transcription [18,19,20,21,22], (iii) affecting translation and/or stability of target mRNAs [2,13,23,24,25], (iv) modulating the function of proteins [18,19,21,26,27,28,29], or (v) by sequestering other sRNAs [30,31,32]. Depending on their mode of action, regulatory RNAs can be classified into seven major subgroups: *s*mall base-pairing *RNAs* (sRNAs), dual-function (bifunctional) RNAs, protein-binding RNAs, RNA sponges, riboswitches, RNA thermometers and CRISPR RNAs. In this review, we focus on base-pairing sRNAs and protein-binding RNAs in the opportunistic human pathogen *Pae*.

## 2. Base-Pairing RNAs in *Pae*

**Figure 1:**
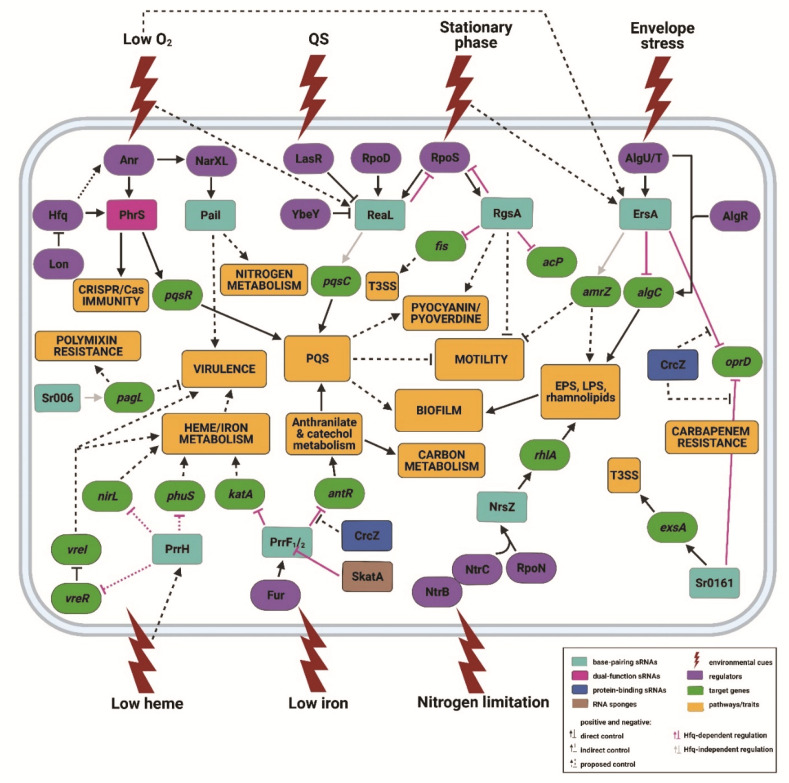
**Regulation by base-pairing sRNAs**. Characterized base-pairing RNAs (malachite green) and dual-function RNA PhrS (magenta) and their respective regulators (violet) and targets (green). The complex RNA-mediated regulatory network governs a number of metabolic and virulence-related processes (orange boxes) in response to different environmental cues (red flashes). See main text for details. ↓: positive control; ┴: negative control; dashed lines: indirect control; dotted lines: proposed control. Light grey lines indicate Hfq-independent regulation, whereas magenta lines highlight Hfq-dependent regulation.

Several different approaches were employed for the identification of sRNAs in *Pae*, including in silico predictions [33,34], co-purification with the RNA chaperone Hfq [35] as well as high-throughput RNAseq under different growth conditions [36,37,38,39]. A total of 573 and 233 sRNAs were detected in strain PAO1 and in the clinical isolate PA14, respectively [37,40,41]. Although 126 sRNAs were identified to be present in both strains, in silico predictions suggest that the actual number of common sRNA is larger [40,41,42]. Only a few of these RNAs have been functionally characterized. These include sRNAs involved in (i) virulence and biofilm formation (ErsA, Sr0161, Sr006, PrrF1/2, PrrH, ReaL, RgsA, Pail, NrsZ), (ii) iron metabolism (PrrF1/2 and PrrH), (iii) nitrogen metabolism (Pail and NrsZ), (iv) quorum sensing (PhrS, PrrF1/2 and ReaL) and (v) antibiotic susceptibility (ErsA, Sr0161 and Sr006) (Figure 1).

RNA chaperones are a special class of RNA-binding proteins fostering the stability and function of sRNAs [43,44,45,46]. In Gram-negative bacteria, including *Pae* [47], the RNA chaperone Hfq (below for further details) can act as an RNA matchmaker, aiding in base-pairing between sRNAs and their target mRNAs, especially when the cognate sRNA/mRNA pair lacks extensive complementarity [48,49].

### 2.1. ErsA

The *e*nvelope stress-*r*esponsive *s*RNA *A* (ErsA) is a *trans*-encoded sRNA which was initially identified in *Pae* strain PAO1 as a functional orthologue of *Escherichia coli* Spot 42 RNA [50]. ErsA was as well recognized in an RNAseq approach in *Pae* strain PA14 as SPA0122 [37]. Transcription of *ersA* is governed by the *e*xtra*c*ytoplasmic *f*unction (ECF) sigma factor AlgU/T (σ^22^), an envelope stress response regulator [51]. The ErsA levels are elevated upon depletion of oxygen and upon entry into stationary phase [51,52]. With the aid of Hfq, ErsA represses the translation of *algC* mRNA (Figure 1), encoding the bifunctional enzyme phosphomannomutase/phosphoglucomutase [51]. AlgC is essential for the biosynthesis of alginate [53,54], the *e*xo*p*oly*s*accharides (EPS) Psl and Pel [55], *l*ipo*p*oly*s*accharide (LPS) [56,57] and rhamnolipids [58], which are components of the biofilm matrix during chronic infection [59,60]. AlgC synthesis is activated by the alginate biosynthesis regulator AlgR and the sigma factor AlgU/T [61,62,63], which together with the sRNA ErsA are part of an incoherent feed-forward loop, which alters the AlgC levels [51]. Apart from its role in the synthesis of biofilm matrix components, ErsA appears to contribute to biofilm development and motility by stimulating the synthesis of the transcriptional regulator AmrZ (Figure 1) [64]. Moreover, ErsA was recently reported to be involved in pathogenesis in both acute and chronic airway infections [65].

The OprD porin serves as an entry port for basic amino acids and peptides, as well as for carbapenem antibiotics [66,67]. Frameshift and nonsense mutations in the *oprD* gene, which prevent synthesis or result in the production of functionally impaired OprD, gave rise to diminished carbapenem susceptibility [68,69,70,71,72,73,74]. A recent GRILseq (*G*lobal sRNA Target *I*dentification by *L*igation and *Seq*uencing) study revealed the *oprD* gene as a novel target of ErsA [52]. The sRNA ErsA negatively regulates *oprD* translation, which in turn leads to decreased OprD levels and thus to reduced meropenem susceptibility (Figure 1) [52].

### 2.2. Sr0161

The sRNA Sr0161 was discovered in *Pae* strain PAO1 by a GRILseq analysis [52]. The *sr0161* gene is located between the genes PA0160 and *opdC*. Functional studies performed by Zhang et al. [53] revealed that Sr0161, like ErsA, regulates the *oprD* gene, and thus carbapenem susceptibility (Figure 1). In addition, Sr0161 was suggested to regulate *opdP* mRNA, which encodes a second porin involved in carbapenem uptake [52]. However, recent studies revealed that *opdP* appears not to be regulated by sRNAs [75]. At variance, *opdP* appeared to be translationally repressed by a regulatory complex consisting of Hfq and the catabolite repression protein Crc (see below). Another target of Sr0161 is the transcriptional activator of the *t*ype *III s*ecretion *s*ystem (T3SS), ExsA (Figure 1) [52]. Sr0161 was shown to bind to the 5′*u*n*t*ranslated *r*egion (UTR) of *exsA* mRNA, and to repress its translation [52]. Hence, the sRNA Sr0161 seems to assist in several virulence-related processes.

### 2.3. Sr006

The sRNA Sr006 was originally annotated during a single nucleotide resolution transcriptome analysis [39]. A GRILseq approach revealed Sr006 as a positive post-transcriptional regulator of the *pagL* gene, encoding the lipid A 3-O deacylase (Figure 1) [52]. The acetylated form of lipid A was previously shown to be pro-inflammatory causing severe pulmonary damage in chronically infected *c*ystic *f*ibrosis (CF) patients [76,77,78]. Sr006 interacts with the 5′UTR of *pagL* mRNA in an Hfq-independent manner [52], resulting in an increase in the PagL levels, which in turn augment lipid A deacetylation of the LPS [76]. As a consequence, polymyxin B susceptibility is elevated [79,80] and pro-inflammatory signalling is decreased [81].

### 2.4. PrrF1/2

The iron-responsive sRNAs PrrF1 and PrrF2 represent functional orthologues of the *E. coli* sRNA RyhB [82] and *Bacillus subtilis* FsrA sRNA [83]. PrrF1 and PrrF2 are encoded in tandem and share >95% sequence identity. The *prrF* genes are transcriptionally controlled by the *f*erric *u*ptake *r*egulator Fur [33]. The PrrF RNAs have been implicated in the maintenance of iron homeostasis under iron-limiting conditions by post-transcriptionally repressing genes encoding non-essential iron-containing proteins (Figure 1) [33,52,84,85,86,87]. PrrF-dependent riboregulation is mediated by Hfq [47]. A GRILseq study identified an RNA sponge derived from the 3′ end of *katA* mRNA, encoding the major catalase in *Pae* [52,86]. The RNA sponge SkatA acts as a decoy for PrrF1, which interferes with PrrF1 base-pairing to the 5′ end of the *katA* transcript, and thus with translational repression [52,86]. Hfq was recently shown to bind to both the SkatA and PrrF RNAs, suggesting a role for their maturation and/or regulation [88].

The PrrF RNAs are further involved in the synthesis of the *Pseudomonas q*uinolone *s*ignal (PQS), a *q*uorum *s*ensing (QS) quinolone affecting early biofilm development and virulence (Figure 1) [89,90,91,92]. The PrrF RNAs impact the expression of the *antABC* and *catBCA* operons, encoding functions required for the catabolism of anthranilate, a precursor of PQS [85]. As a consequence, anthranilate is re-directed into the PQS anabolic pathway [85]. In addition, the PrrF sRNAs were shown to foster the synthesis of other 2-*a*kyl-4-*q*uinolones (AQs), which are contributing to virulence [93]. The promoting effect on AQs was attributed to PrrF/Hfq-mediated repression of *antR* mRNA, encoding a transcriptional activator of the *antABC* and *catBCA* operons [47,85,94]. Thus, the Prrf-dependent regulation of AQ biosynthesis represents an example of a crosstalk between iron, carbon metabolism and virulence in *Pae*. In addition, a recent proteomic study further expanded the PrrF-dependent regulon for several iron-regulated proteins, which are involved in amino acid metabolism, twitching motility and zinc homeostasis [95].

### 2.5. PrrH

The heme-responsive sRNA PrrH is transcribed from the tandem *prrF1*/*prrF2* locus through heme-dependent anti-termination of *prrF1* transcription [96]. The longer PrrH RNA binds to Hfq with lower affinity than the PrrF RNAs [97]. PrrH is deemed to fine-tune the expression of genes required for heme uptake and metabolism (Figure 1) [96]. For instance, *nirL* mRNA, which was described as a putative target of PrrH RNA [96], is part of the *nirSMCFDLGHJEN* operon, encoding the dissimilatory nitrite reductase NirS (cytochrome *cd*_1_). The *nir* operon also contains genes of the heme *d*_1_ biosynthetic pathway, which is an alternative branch of the central heme biosynthetic pathway [98]. Thus, the predicted PrrH-mediated repression of the alternative Nir heme *d*_1_ biosynthesis under heme-limiting conditions may favor the activity of the central heme biosynthetic pathway. Moreover, PrrH is predicted to regulate *phuS*, the product of which is responsible for the flux of heme through the heme-oxygenase HemO [99,100]. Heme represents an abundant source of iron in the human host. Therefore, the ability of the bacterium to assimilate and utilize heme might provide a competitive advantage during infection. In silico analyses performed to reveal potential virulence targets for PrrH identified *vreR* as a putative candidate [99]. VreR is an anti-sigma factor of the cell surface signalling ECF sigma factor VreI, and is thought to be involved in *Pae* virulence [101,102,103]. The translational start site and coding sequence of *vreR* mRNA share extensive complementarity with PrrH [99]. Moreover, the deletion of the *prrF* locus leads to a threefold increase in *vreR* expression, suggesting a potential role of PrrF/PrrH in its regulation [99].

### 2.6. ReaL

The highly conserved sRNA ReaL (*re*gulator of *a*lkyl quino*l*one) was initially identified in strain PAO1 as PaeIII [104], and as SPA0084 in strain PA14 [37]. The ReaL levels are elevated during stationary phase, as well as under anoxic conditions. ReaL transcription is governed by the “house-keeping” sigma factor RpoD (σ^70^) and by the stationary phase sigma factor RpoS (σ^38^, σ^S^) [105]. Moreover, ReaL impedes translation of *rpoS* mRNA through base-pairing, thus imposing negative feedback control (Figure 1) [106]. An additional layer of regulation in the ReaL-RpoS negative feedback loop was recently proposed. The endoribonuclease YbeY was implicated in the regulation of *rpoS* through degradation of ReaL [107].

ReaL is also involved in QS regulation, connecting the Las and PQS systems in *Pae* [105]. Las and PQS, together with the *i*ntegrated *q*uorum *s*ensing system (IQS) and the Rhl QS system, represent a hierarchically organized regulatory quorum sensing network, which mediates *Pae* virulence during infection [108]. The PQS system is essential for shaping a more stress-tolerant bacterial population in response to harsh environmental conditions [109,110]. Moreover, PQS synthesis results in elevated biofilm formation, pyocyanin and pyoverdine synthesis [111,112,113], and in decreased swarming [113]. These phenotypes are commonly detected during the colonization of CF lungs [113,114,115]. PQS biosynthesis requires the functions encoded by the *pqsABCDE* and *phnAB* operons, as well as the *pqsH* gene [116]. Previous reports linked LasR, the transcriptional regulator of the Las QS system, to PQS biosynthesis control [89]. *L*iquid *c*hromatography coupled to *m*ass *s*pectrometry (LC/MS) analyses [117] demonstrated diminished levels of PQS and accumulation of its precursor 4-*h*ydroxy-2-*h*eptyl*q*uinoline (HHQ) in a *lasR* deletion strain, implicating LasR in HHQ to PQS conversion [118,119]. Moreover, LasR seems to directly activate *pqsH* and *pqsR* expression [116,120,121,122]. However, LasR also inhibits *reaL* transcription [105]. ReaL activates *pqsC* translation in an Hfq-independent manner, and in turn elicits elevated PQS levels and promotes establishment of PQS-dependent virulence traits (Figure 1) [105]. Therefore, LasR inhibition of *reaL* expression leads to diminished PQS levels and impaired PQS-related phenotypes [105]. Hence, ReaL acts as a modulator of LasR-dependent PQS biosynthesis and virulence.

### 2.7. RgsA

The sRNA RgsA was identified by two independent bioinformatic analyses in strain PAO1 [34,123]. Synthesis of RgsA is induced upon shift to stationary phase, and is dependent on the stationary phase sigma factor RpoS [123]. Transcription of *rgsA* is also indirectly activated by the GacA/S *t*wo-*c*omponent *s*ystem (TCS) [123,124]. In addition, RgsA requires Hfq for its stability [124,125]. Initial results suggested that RgsA might contribute to the oxidative stress response [123,126], while another study by Lu et al. (2016) implicated RgsA in pyocyanin synthesis and swarming motility. RgsA was shown to inhibit translation of the *fis* and *acpP* mRNAs, encoding the global transcriptional regulator Fis and the acyl carrier protein AcpP, respectively, in an Hfq-dependent manner (Figure 1) [124]. Fis is a DNA-binding and bending protein [127,128], which is essential for the expression of the *t*T3SS and T3SS-mediated cytotoxicity in *Pae* [129], and was recently deemed to contribute to ciprofloxacin resistance [130].

### 2.8. PaiI

The highly conserved sRNA PaiI (*P*seudomonas *a*naerobically *i*nduced RNA I) was identified in the intergenic region between the genes PA14_13970 and PA14_13990 in strain PA14 [131]. It was shown to be abundant under anoxic conditions, and rapidly degraded upon a shift to aerobic conditions [131]. PaiI transcription is induced during anaerobic denitrification, and is governed by the nitrate-responsive TCS NarL/X [131], whose expression is under control of the anaerobic regulator Anr [132]. Further studies revealed that PaiI is necessary for anaerobic growth of *Pae* on glucose in the presence of nitrate [131]. PaiI was also demonstrated to impact the conversion of nitrite to nitric oxide during denitrification. However, the impact on denitrification seems to be a result of indirect effect(s) on the activity of the nitrite reductase NirS [131]. In addition, the absence of PaiI resulted in impaired growth of the mutant in an in vivo CF lung infection model, highlighting the potential importance of PaiI for adaptation of *Pae* under these conditions [131].

### 2.9. NrsZ

NrsZ represents the first reported nitrogen-regulated sRNA in PAO1, which is highly conserved among *Pseudomonads* [133]. It is transcribed from the *ntrC*-PA5126 intergenic region under nitrogen-limiting conditions by the joint action of the nitrogen-responsive TCS NtrB/C and the sigma factor RpoN (σ^54^, σ^N^) [133]. Although the NrsZ full-length transcript is ≈226 nt long, the most abundant detected species are ≈40 nt and 140 nt in length [133]. Moreover, a processed transcript, encompassing the first 60 nt, was shown to be functional in riboregulation [133]. Thus, processing of NrsZ RNA is probably required for its activity, as it is the case with some other sRNAs such as ArcZ [134] and MicX [135]. In *Pae*, NrsZ promotes swarming motility by translational activation of the *rhlA* gene (Figure 1), the product of which is required for rhamnolipids biosynthesis [133]. Rhamnolipid biosurfactants, together with flagella, are necessary for swarming of *Pae* [136]. NrsZ appears to interact with an intramolecular secondary structure in the *rhlA* 5′UTR comprising the *r*ibosome *b*inding *s*ite (rbs) [133]. This interaction might melt the inhibitory structure and thereby promote translation of *rhlA* mRNA [133].

### 2.10. PhrS

The PhrS RNA represents the first described anaerobically controlled sRNA in *Pae* [137]. PhrS synthesis is induced under hypoxic conditions [35], and requires the anaerobic regulator Anr [137]. PhrS expression is also affected by Hfq, most probably indirectly *via* Hfq-mediated regulation of Anr [137]. PhrS was shown to stimulate pyocyanin biosynthesis by increasing the production of PQS [137]. PhrS regulates the synthesis of PQS by indirectly activating the translation of *pqsR* mRNA, encoding the QS regulator PqsR (MvfR) (Figure 1) [137]. PqsR acts as an activator of the *pqsABCDE* operon, required for biosynthesis of PQS [138]. The mechanism of PhrS-dependent translational activation of the *pqsR* transcript includes translational coupling, whereby the translation of *pqsR* mRNA is positively coupled with the translation of an *u*pstream *o*pen reading *f*rame (*uof*)*,* the translation of which is stimulated by PhrS [137]. The PhrS base pairs to and resolves an inhibitory secondary structure that occludes the rbs of *uof*, and thereby activates the translation of *uof*, and thus of *pqsR* mRNA [137].

Fernandez et al. [139] provided evidence for a role of PhrS in the regulation of swarming and biofilm formation. The authors observed enhanced swarming and increased biofilm biomass when the levels of PhrS were elevated. The increased levels of PhrS were in turn reconciled with elevated Hfq levels due to decreased synthesis of the Hfq-degrading protease Lon under the tested conditions [140].

A recent study proposed a novel function for PhrS as a positive regulator of CRISPR-Cas immunity (Figure 1) [141]. Here, PhrS is deemed to act as a transcriptional anti-terminator of Rho-dependent termination by binding to the CRISPR leaders [141]. This would lead to the transcription of the CRISPR locus and synthesis of CRISPR RNA (crRNA), and thus to adaptive immunity [141].

Apart from its role as a regulatory RNA, PhrS encodes a highly conserved small 37 aa peptide [35,137], and thus is regarded as a dual-function sRNA. However, the function of the peptide has so far remained elusive.

## 3. Translational Repressors and Their RNA Decoys

**Figure 2:**
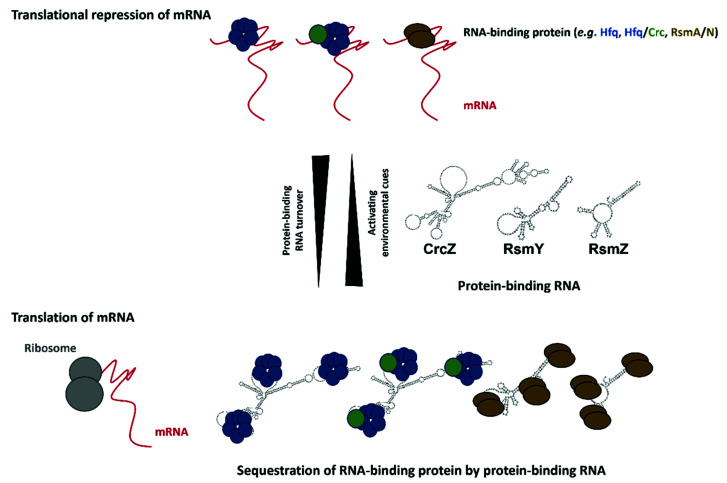
**Regulation by protein-binding RNAs.** Protein-binding RNAs (e.g., CrcZ, RsmY/Z) are activated by environmental cues. They contain multiple binding sites for RNA-binding translational repressors (**top**; Hfq (blue hexamer), Hfq in conjunction with Crc (blue hexamer/green circle), RsmA/N (brown circles)). This leads to sequestration of the RNA-binding proteins and to de-repression of their target mRNAs (**bottom**).

*Pae* features two comprehensive post-transcriptional networks, which are governed by Hfq/Crc [29,142] and the CsrA-like Rsm proteins [142,143], respectively, and their antagonizing RNAs (Figure 2).

**Figure 3:**
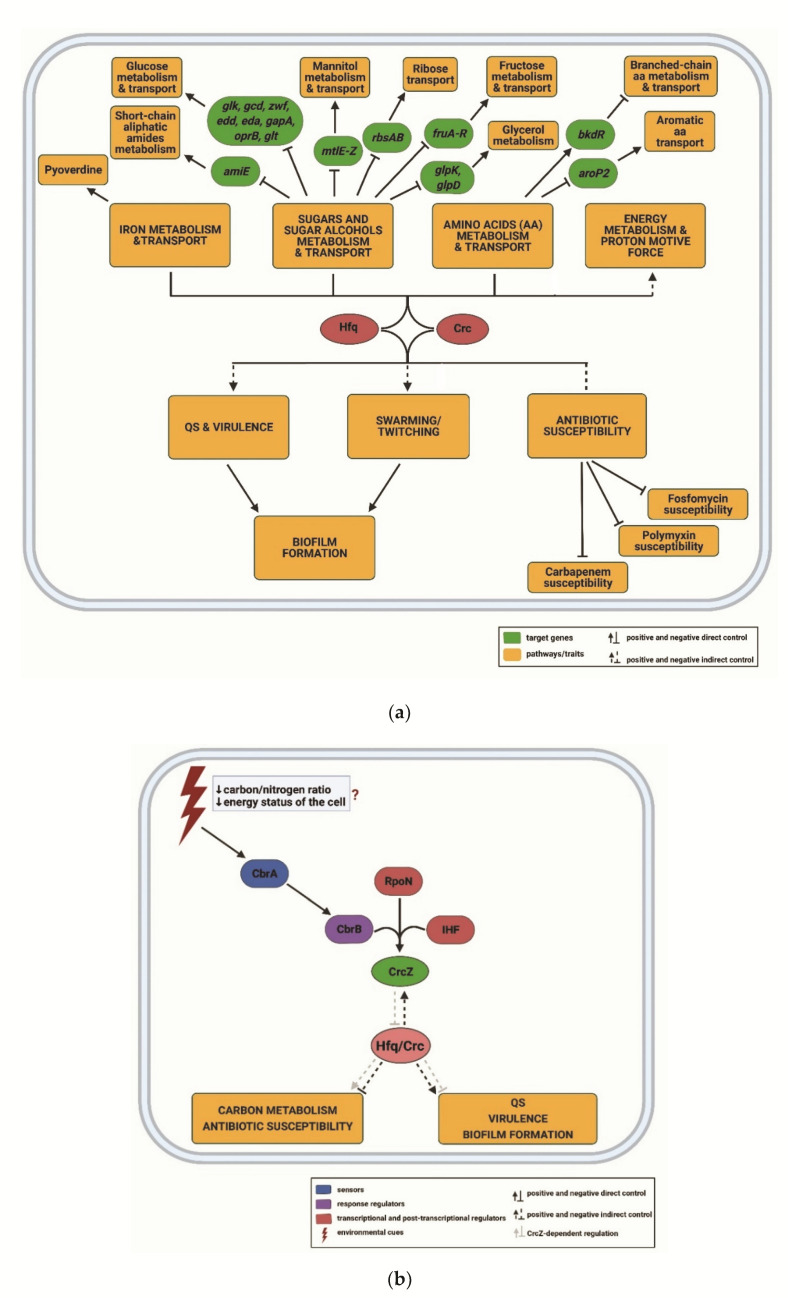
(**a**) **The Hfq/Crc regulon.** Hfq and Crc can exert translational repression of genes involved in carbon, iron and energy metabolism (top). In addition, they are implicated in direct (straight lines) or indirect (dashed lines) regulation of QS, virulence, antibiotic susceptibility and biofilm formation (bottom). The scheme was drawn based on previous reports [142,147,150,170,171,172]. (**b**) **Transcription and stability of CrcZ RNA.** The CrcZ RNA can compete for Hfq and/or Hfq/Crc repressive complexes, and thereby cross-regulate physiological processes involved in carbon metabolism and virulence (light grey lines; (**a**)). The synthesis of CrcZ RNA occurs under energy-limiting conditions in the presence of an unfavorable carbon source by the joint action of the CbrA/B TCS, which is activated by a yet unknown signal (red question mark), IHF and sigma factor RpoN, ↓: positive control; ┴: negative control.

Hfq belongs to the Sm/Lsm family of RNA-binding proteins, ubiquitously present in Eukaryotes, as well as in many Eubacteria and Archaea [144,145]. It acts as a pleiotropic post-transcriptional regulator, which, in *Pseudomonads*, can act in concert with the *c*atabolite *r*epression *c*ontrol protein Crc [142,146,147,148]. Hfq-dependent regulation controls metabolism [29] and virulence traits [47,51,124,149,150,151], including the susceptibility to clinically relevant antibiotics (Figure 3a) [52,75,152]. As mentioned above, Hfq can exert these functions by aiding riboregulation by sRNAs [47,51,52,153], but also by directly repressing the translation of target mRNAs containing cognate Hfq-binding motifs [29,151].

Many sRNAs bind to the proximal side of Hfq through either an AU_5_G-rich sequence [154,155,156,157] or *via* polyU-tails of their rho-independent terminators [158,159,160]. Another RNA-binding surface consists of a basic arginine rich-patch located on the lateral rim of Hfq, which was found to interact with internal U/A-rich regions of sRNAs and mRNAs [49,161,162,163,164]. Moreover, RNAs can associate with the distal binding surface of Hfq [29,148,157,165,166,167] *via* AA(R)N triplets, wherein A is an adenine, R is a purine and N is any nucleotide. Although the A-rich stretches are predominantly found in mRNAs, several studies have shown that some regulatory RNAs also contain A-rich sequences which enable interaction with the distal surface of Hfq [29,164,168,169]. In fact, the repressive function of Hfq can be antagonized through sequestration by the regulatory RNA CrcZ, containing multiple A-rich motifs, which in turn results in de-repression of Hfq and Hfq/Crc target genes (Figure 2 and Figure 3b).

**Figure 4:**
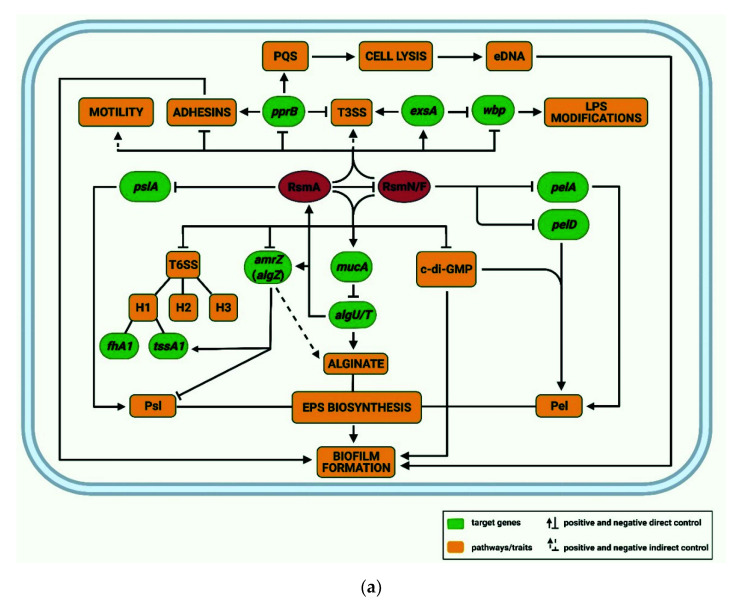

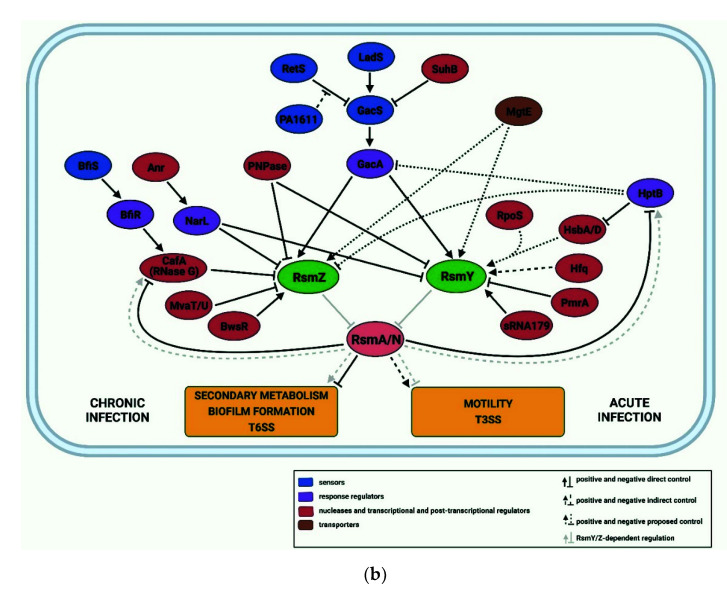
(**a**) **The RsmA and RsmN/F regulons.** RsmA and RsmN/F proteins can act as direct or indirect repressors or activators of different target mRNAs (in green) and pathways (in yellow), which contribute to the shift between the acute (planktonic) and the chronic (sessile, biofilm) lifestyle. RsmN/F protein synthesis is negatively controlled by RsmA at the translational level. The scheme was drawn based on previous reports [139,143,184]. (**b**) **Synthesis** **and turnover of RsmY/Z RNAs.** The RsmY and RsmZ RNAs serve as molecular decoys for the RsmA/N proteins, which in turn mediate the transition from an acute to the chronic infection phenotype (**a**). Transcription and turnover of RsmZ and RsmY are under the control of several two-component systems (sensors: blue ovals; response-regulators: violet ovals), transporters (brown ovals), nucleases and transcriptional and post-transcriptional regulators (dark red ovals), which act either in a GacA/S-dependent (e.g., SuhB-mediated repression of RsmY/Z synthesis) or independent manner (e.g., Anr-mediated repression of RsmY/Z expression). Some regulators affect the abundance of both RNAs (e.g., GacA/S transcriptional activation of RsmY/Z), whereas some are specific for only one of the RNAs (e.g., BfiR-dependent CafA degradation of RsmZ and Hfq-dependent stabilization of RsmY). The complex regulation of Rsm RNAs enables fine-tuning of the expression of RsmA/N targets in response to different environmental cues. Dashed lines: indirect control; dotted lines: proposed mechanism. Light grey lines indicate RsmY/Z-dependent regulation. Figure adapted from [188]. ↓: positive control; ┴: negative control.

The CsrA/RsmA family of RNA-binding proteins governs virulence gene expression in pathogenic bacteria [173,174,175,176,177,178], including *Pae* [139,179]. Members of this family usually negatively regulate translation of target mRNAs through binding to stem-loop structures present in their 5′UTR that contain exposed GGA motifs [27,139,173,180,181,182,183,184]. Rsm proteins can also act as indirect activators and repressors of a large subset of genes by altering the expression of key regulators (Figure 4a) [139,179,185]. Moreover, Rsm proteins can stabilize transcripts, and thereby promote their translation [184,186,187]. Analogously to the Hfq/Crc/CrcZ entity, the activity of these translational regulators can be countered by RsmA/N-binding RNAs, which act as a decoy to abrogate RsmA/N-mediated translational repression and thus, lead to de-repression of RsmA/N controlled genes (Figure 2 and Figure 4b).

### 3.1. The Hfq Regulon

In *Pseudomonas*, the hierarchical assimilation of carbon sources is governed by the RNA chaperone Hfq [29] and the *c*atabolite *r*epression *c*ontrol protein Crc [146,170,171,172,189,190,191,192,193,194,195,196] (Figure 3a). Under conditions of *c*arbon *c*atabolite *r*epression (CCR), Hfq and Crc form a repressive complex on target mRNAs, the encoded proteins of which are involved in uptake and/or utilization of carbon and nitrogen sources other than the preferred one [29,147,197]. In the repressive complex, Crc acts as a translational co-repressor that promotes Hfq-mediated translational repression of target mRNAs [29] by binding to Hfq/mRNA complexes [147,148], thereby increasing stable Hfq/Crc/RNA complex formation [198]. A recent ChIP-seq analysis and a combined RNA-seq/proteomics study identified 100 [142] and 244 mRNAs [172], respectively, to be co-regulated by Hfq and Crc. Almost all of the nascent transcripts binding to Crc were also found to be associated with Hfq, emphasizing the auxiliary role of Crc in Hfq-dependent regulation [142].

Several transcriptome, proteome and metabolome analyses have implicated Hfq and Crc in the regulation of iron metabolism and transport [150,171], as well as in energy metabolism and generation of the *p*roton *m*otive *f*orce (PMF) [150,152,170,171,196,199] (Figure 3a). Furthermore, several studies have linked Hfq with the activation of QS regulators [147,149,150]. A PAO1 Δ*hfq* deletion strain showed an increased pyocyanin production [137,149,150] and an attenuated virulence phenotype [149,150], the latter of which can be explained by an impairment in swarming, twitching [140] and biofilm formation [199]. Moreover, the Hfq deficiency caused an increased susceptibility to different classes of antibiotics [152] (Figure 3a). The same phenotypes were observed with a Δ*crc* deletion strain [147,151,170,171,194,200,201] (Figure 3a). Many of these effects are probably indirect and are caused by de-regulation of transcription regulators and/or of the quantities of metabolites, which can act as transcription effectors [172]. However, a recent ChIPseq analysis has also provided evidence for direct regulation of some of the pathways by Hfq/Crc [142].

### 3.2. CrcZ, a Decoy for Hfq

As mentioned above, Hfq/Crc-mediated regulation is antagonized by the Hfq-binding RNA CrcZ [29,147,193]. The CrcZ RNA is highly conserved in the genus *Pseudomonas* [202], and some *Pseudomonas* species contain additional genetically and/or functionally redundant CrcZ RNA homologs such as CrcY and CrcX in *P. syringae* [203,204], and CrcY in *P. putida* [202] and *P. fluorescens* [202,205], respectively. The CrcZ RNA and its homologs contain several A-rich motifs, which can interact with the distal RNA-binding surface of Hfq [29,88,193,197,205]. For instance, Hfq has a ~5–20-fold higher affinity for CrcZ than for its target *amiE* mRNA, encoding an aliphatic amidase [29]. Hence, the sequestration of Hfq by CrcZ/Y prevents Hfq from binding to target mRNAs and results in translational de-repression [29,147,196,197,205]. In addition, through the competition for Hfq, CrcZ can also interfere with Hfq-dependent riboregulation by sRNAs [47] and hence, indirectly cross-regulate other physiologically important processes such as biofilm formation [199] and antibiotic susceptibility [75,152] (Figure 3).

In PAO1, the 407 nt long CrcZ RNA is encoded in the *cbrB-pcnB* intergenic region [193]. The *crcZ* gene is transcribed from an RpoN-dependent promoter with the aid of the response regulator CbrB and *i*ntegration *h*ost *f*actor (IHF) (Figure 3b) [193,206]. CbrB together with its cognate histidine kinase CbrA forms a TCS involved in the maintenance of carbon and nitrogen balance [207,208]. CbrA/B is activated by an unknown signal, which might depend on the energy status of the cell [209]. The CbrA/B activity and the resulting CrcZ levels depend on a given carbon source [193,209]. For instance, the CrcZ levels are low during fast growth in the presence of a preferred carbon source, e.g., succinate [193,209], whereas CrcZ is highly abundant during slow growth in the presence of unfavorable carbon sources, e.g., oxaloacetate [209]. Hernandez-Arranz et al. [210] demonstrated that the *crcZ* expression can also be driven from the weak constitutive promoter of the *cbrB* gene resulting in a *cbrB-crcZ* transcript, which is subsequently processed to yield the ≈407 nt long CrcZ RNA. In addition, several shorter variants of CrcZ seem to result from either premature termination of transcription or from degradation by a yet unknown nuclease [193]. It was further shown that Hfq in concert with Crc affects both the transcription and stability of CrcZ in *P. putida* [211].

### 3.3. The RsmA and RsmN/F Regulons

The RNA-binding proteins RsmA [212] and RsmN/F [182,213] represent two orthologs of the well-studied *c*arbon *s*torage *r*egulator CsrA, which primarily serves as a translational repressor and pleiotropic regulator of various processes in *E. coli* [28,214,215,216,217]. RsmA and RsmN/F act in concert and have overlapping as well as unique regulatory roles (Figure 4a) [139,182,218]. Although the absence of RsmN/F has little or no impact on the expression of RsmA-regulated genes, the deletion of both *rsmA* and *rsmN/F* causes additional phenotypes when compared with a single *rsmA* deletion [182,218]. In addition, RsmN/F and RsmA exhibit distinct binding affinities for some of their RNA targets [142,182,183,184,218,219]. RsmN/F binds only to targets that contain at least two Rsm consensus sequences, while RsmA requires only a single GGA motif for efficient interactions [183,184]. Moreover, RsmA functions as a translational repressor of RsmN/F (Figure 4a) [182]. Thus, RsmN/F seems to play an auxiliary role in the hierarchical Rsm regulatory cascade (Figure 4a).

The Rsm proteins govern the synthesis of many proteins involved in the transition between acute and chronic virulence phenotypes [27,139,143,174,179,182,184,185,220] (Figure 4a). For instance, they control virulence traits associated with acute infection such as the T3SS, motility and LPS modifications (Figure 4a) [139,143,184,185]. The effect of Rsm proteins on T3SS expression seems to occur through positive regulation of T3SS transcription activators [221], such as ExsA [185,222,223], and/or by inhibition of the T3SS negative regulator PprB [139,224] (Figure 4a). The Rsm-mediated regulation of motility is mainly associated with modulation of flagella and type IV pili biosynthesis [185], which appears to be indirect through the regulation of other transcriptional regulators such as AmrZ [64,143,185,225,226]. Moreover, AmrZ serves as an activator for the synthesis of the *t*ype *VI s*ecretion *s*ystem (T6SS) [143,227], alginate biosynthesis [228,229,230,231] and biofilm development [232]. Vice versa, it functions as an inhibitor of flagellum synthesis [233] and exopolysaccharide Psl production [234] (Figure 4a). A recent study [185] has linked Rsm-mediated regulation with O-antigen biosynthesis and LPS modification. The *wzx*, *wzy* and *wzz* genes, the products of which are involved in regulation and assembly of the O-antigen, and the *wbp* operon, which is important for the biosynthesis and assembly of the nucleotide sugars of the O-unit [235], were found to be translationally repressed by RsmA/N (Figure 4a) [184]. This, in turn, can lead to the LPS-rough phenotype, often present in *Pae* isolates during chronic CF lung infections [59]. Moreover, the *wzz2* expression can be transcriptionally repressed by AmrZ [236], whereas the expression of *wbpA* and *wbpH* genes appears to be directly governed by the T3SS transcription regulator ExsA [237] (Figure 4a). Hence, the O-antigen biosynthesis and LPS modification seems to be under both direct translational and indirect transcriptional control of Rsm proteins.

Besides their positive impact on acute virulence traits, the Rsm proteins also negatively impact features important in chronic infections, e.g., T6SS synthesis, EPS production, c-di-GMP signaling and biofilm formation [185,238,239,240,241,242] (Figure 4a). RsmA/N serve as translational repressors of several T6SS genes. Among them, the effect on the expression of *tssA1* and *fha1* T6SS structural genes is well studied [143,182,185,242]. The RsmA/N-mediated repression of *amrZ* does not only impact motility as discussed above, but also EPS [139,143,227,232] and T6SS biosynthesis [228,229,234] (Figure 4a). Moreover, RsmA/N can directly inhibit the synthesis of EPS (Figure 4a). For instance, RsmA translationally represses *pslA* mRNA [243], whereas RsmN/F was shown to inhibit the synthesis of PelA and PelD [139]. Both Rsm proteins promote the expression of the *mucA* gene, probably by binding to and stabilizing its transcript [139]. MucA encodes an anti-sigma factor of the alternative sigma factor AlgU/T, which, in addition to its role in envelope stress response, acts as a key regulator in alginate biosynthesis [244,245]. Furthermore, previous studies have implicated AlgU in promotion of *rsmA* [246] and *amrZ* transcription [228], respectively (Figure 4a). In addition to the inhibition of EPS biosynthesis, which plays an important role in biofilm formation, Rsm proteins mediate the repression of other biofilm-related pathways and regulators (Figure 4a). As mentioned above, RsmN/F inhibits the expression of *pelD* [139], which is involved in c-di-GMP-dependent regulation of Pel biosynthesis [247] (Figure 4a). The c-di-GMP signaling and Rsm regulatory network were shown to be intertwined and antagonistically regulate the shift from the motile to the sessile phenotype [248,249,250,251].

Another direct target of Rsm proteins is the response regulator PprB [139], which is not only known as a negative regulator of T3SS, but also as a positive regulator of adhesins [224], PQS-mediated cell lysis and of the release of *e*xtracellular *DNA* (eDNA), which contribute to biofilm formation [252] (Figure 4a).

Last but not least, Rsm proteins appear to modulate the expression of several transcripts encoding *p*hospho*d*i*e*sterases (PDEs) and *d*i*g*uanylate *c*yclases (DGCs), which impact the c-di-GMP levels, which in turn widely affects gene expression and function [139,250].

### 3.4. RsmV/W/Y/Z, Decoys for RsmA/N/F

The function of the Rsm proteins is controlled by Rsm-binding RNAs, which act by titration [28,184,186,218,219,253]. Four RsmA- and RsmN/F-titrating RNAs, RsmZ/B [186,254], RsmY [150,255], RsmW [256] and RsmV [219], have been described in *Pseudomonads*. The Rsm RNAs contain multiple GGA motifs, which serve as Rsm binding sites [27,186,218,219,253,255,256,257]. Different binding affinities of Rsm proteins for Rsm RNAs are deemed to provide a mechanism of modulating the Rsm regulatory network in response to environmental signals [218]. The sequestration of the Rsm proteins by the Rsm RNAs leads to the transition from a planktonic/acute infection phenotype to a biofilm/chronic infection phenotype (Figure 4b). At the molecular level, the transition results in differential expression of various genes including those encoding components of the T3SS and the T6SS, respectively (Figure 4b) [27,139,143,179,182,184,185,188,218,219,223,243,256,258,259,260,261].

RsmY and RsmZ are the main RsmA/N/F binding RNAs [27,185,218], while RsmV and RsmW seem to contribute to the dosage effect and temporal modulation of the RsmA/RsmN/F regulatory cascade in response to specific environmental signals in strain PAO1 [218,219,256]. Each Rsm RNA displays different and unique expression patterns and mechanisms for turnover and stability. For instance, the synthesis of RsmY and RsmZ is transiently elevated in late logarithmic growth phase, and then significantly increases in late stationary phase, whereas onset of *rsmW* transcription was observed in early stationary phase [219]. On the contrary, the steady-state levels of RsmV are steadily increasing throughout growth [219]. Moreover, *rsmW, rsmY* and *rsmZ* expression is elevated in biofilms [256,262]. However, RsmZ appears to repress early biofilm formation [253], highlighting the importance of controlled fine-tuning of the Rsm RNA levels [262].

The differential expression patterns of the Rsm RNAs can be attributed to distinct mechanisms of their transcription and turnover. Whereas the transcription of *rsmY* and *rsmZ* is directly controlled by the TCS GacA/S (Figure 4b) [253,259] and by the two orphan sensor kinases, LadS and RetS (Figure 4b) [238,263], the expression of *rsmW* and *rsmV* does not directly depend on the GacA/S-system [219,256]. A yet unidentified signal triggers auto-phosphorylation and the activation of the sensor kinase GacS, which in turn phosphorylates the response regulator GacA. The activity of GacS is under the control of RetS and LadS [238,263,264,265]. The sensor kinase RetS directly binds to GacS and impedes its auto-phosphorylation, and thus prevents subsequent activation of the response regulator GacA [266]. In contrast, the orphan kinase LadS activates the response regulator GacA by phosphorylation [267]. Thus, RetS and LadS are antagonistically driving the expression of GacA/S-dependent genes, enabling the transition between acute and chronic infection phenotypes [263]. Another study revealed that the hybrid sensor kinase PA1611 binds to RetS and interferes with its binding to GacS (Figure 4b), and thus indirectly alters the synthesis of RsmY and RsmZ RNAs [268]. Furthermore, the hybrid sensor kinase HptB was shown to negatively control *rsmY* expression in planktonic cells in a GacA/S-dependent manner [260,269]. Recently, it was demonstrated that during stationary phase, HptB can also alter the synthesis of RsmY independently of the GacA/S regulatory cascade *via* an HsbA/D and RpoS-mediated pathway (Figure 4b) [270]. Moreover, HptB also inhibits *rsmZ* transcription in a GacA/S-independent way in swarming cells (Figure 4b) [269]. Similarly, the ribosome-associated protein SuhB, which is deemed to promote synthesis of T3SS and to inhibit synthesis of T6SS, indirectly inhibits the production of RsmY and RsmZ by repressing *gacA* expression (Figure 4b) [271].

The transcription of *rsmZ* and *rsmY* is further under control of several additional transcription regulators (e.g., Anr-mediated NarL repression of *rsmY*/*Z* expression; [272]) and transporters (e.g., MgtE-dependent activation of *rsmY*/*Z* transcription; [273]), which, in a GacA/S-independent manner, can activate or repress T3SS (Figure 4b).

In contrast to the GacA/S transcriptional activation of both RsmY and RsmZ, there are some regulatory pathways which specifically modulate the expression of only one of the RNAs in PAO1. Examples include sRNA179-mediated RsmY synthesis [274] and PmrA-mediated inhibition of *rsmY* transcription [275] (Figure 4b), which specifically effect only the expression of RsmY, but not RsmZ. Furthermore, the histone-like nucleoid structuring proteins MvaT and MvaU were previously shown to inhibit *rsmZ* transcription by binding to AT-rich region upstream of the *rsmZ* promoter (Figure 4b) [259,276]. The inhibitory effects of MvaT/MvaU are in turn counteracted by the transcriptional regulator BswR (Figure 4b) [277].

The stability and turnover of RsmZ and RsmY RNAs in strain PAO1 is modulated by several regulators, which can affect the stability of both RNAs (e.g., RsmA-dependent stabilization of RsmY and RsmZ; Figure 4b; [253,278]). On the other hand, the BfiR/S-dependent CafA (RNase G) degradation affects only RsmZ during biofilm development [262] and RsmY RNA appears to be stabilized by Hfq [150,279] (Figure 4b). In addition, polynucleotide phosphorylase (PNPase), a phosphate-dependent 3′–5′ exonuclease previously linked to virulence and bacterial stress response [280], was reported to regulate multiple virulence factors by affecting RsmY and RsmZ turnover [281]. It was recently demonstrated that RsmA and RsmN/F proteins can also alter *rsmZ* and *rsmY* expression indirectly by interfering with RNase G (CafA) and HptB/HsbA/D synthesis, respectively [185] (Figure 4b).

Taken together, the transcription as well as the stability/levels of Rsm titrating RNAs are governed by numerous regulatory circuits responding to environmental cues as well as to the physiological state of the cells, which not only permits to mount an acute or a chronic infection phenotype but probably also to fine-tune their formation.

## 4. Perspectives

Many candidate regulatory RNAs have been revealed in bacteria owing to the development of novel high-throughput technologies. Recent examples include *grad*ient profiling by *seq*uencing (Gradseq) [282,283] or *R*Nase-sensitive *grad*ient profiling by mass spectrometry (GradR) [284] for the discovery of RNA/protein complexomes [284,285,286,287,288]. The resulting follow-up studies will certainly advance our understanding of intricate regulatory RNA-protein interacting networks in bacteria.

Unravelling the RNA-based regulation of processes controlling metabolism, cell growth, biofilm formation, antibiotic susceptibility and other virulence traits of (multi)drug-resistant pathogens such as the ESKAPE class (***E****nterococcus faecium*, ***S****taphylococcus aureus*, ***K****lebsiella pneumoniae*, ***A****cinetobacter baumannii*, ***P****seudomonas aeruginosa*, and ***E****nterobacter* species) [289,290] will be rewarding for the development of novel antimicrobials [291,292]. Such attempts are exemplified by the development of anti-sense oligomers, especially *p*eptide *n*ucleic *a*cids (PNAs), which can act as precision antimicrobials selectively targeting species-specific mRNAs under certain conditions [293,294,295,296,297,298,299]. PNAs and other anti-sense oligomers often show synergistic effects with existing antibiotics [300,301,302,303]. Hence, these novel insights might change the treatment of infections caused by (multi)drug-resistant pathogens [304,305,306] as well as by multi-species [307] during acute and chronic infections [303,308].

Moreover, the discovery of new RNA chaperones such as ProQ [282,309,310,311,312,313,314], as well as novel functions of well-known RNA-binding proteins [315,316], show that the complexity of RNA-based regulation is increasing. Given the small number of characterized bacterial regulatory RNAs and their protein chaperones, further endeavors are required to understand RNA-mediated regulatory circuits. The continuous development of new technology to study RNA-mediated processes in conjunction with conventional biochemical and genetic approaches will certainly foster further exciting insights into RNA-based regulation in bacteria, including bacterial pathogens such as *Pae*.
